# The regulation of mobile health applications

**DOI:** 10.1186/1741-7015-10-46

**Published:** 2012-05-08

**Authors:** Amy J Barton

**Affiliations:** 1 University of Colorado, Anschutz Medical Campus, College of Nursing, 13120 East 19th Avenue, Aurora, Colorado 80045, USA

**Keywords:** Mobile medical applications, Regulation, Mobile health

## Abstract

In July 2011, the United States Food and Drug Administration issued draft guidance concerning the regulation of mobile medical applications (applications on a wireless device that are used as accessories to medical devices or to convert a mobile platform to a medical device). While the suggestion of regulation is rooted in patient safety, concerns about limits on innovation and discovery as well as the evolving nature of both mobile health and current healthcare delivery have emerged. This article discusses the prevalence of mobile health, the context of regulation concerning mobile medical applications, and implications for the future.

## Introduction

Mobile health, or more commonly, mHealth, is 'the use of wireless communication devices to support public health and clinical practice' [1]. Mobile devices are handheld in nature and include mobile phones, personal digital assistants, patient monitoring devices, and other wireless devices. mHealth applications are receiving increased attention largely due to the global penetration of mobile technologies. It is estimated that over 85% of the world's population is now covered by a commercial wireless signal, with over 5 billion mobile phone subscriptions [2]. The availability of mobile technology has advanced infrastructure development in low- and middle-income countries beyond roads and electricity [2]. Mobile medical applications range from communication between individuals and health systems (such as call centers, appointment reminders, treatment compliance) to health monitoring and surveillance (including surveys, patient monitoring devices), and access to information at the point of care (health records, decision support).

The Royal Tropical Institute [3] defined eight mHealth application areas. Education and awareness systems are those that provide information about health promotion and disease prevention. Point-of-care support and diagnostics are used to provide the clinician with reference information for clinical care as well as decision support for diagnostics. Patient monitoring provides patients with support for treatment adherence. Disease and epidemic outbreak surveillance provides real-time tracking of infectious diseases. Emergency medical response systems provide alerts for accidents and disasters. Health information systems manage data used in clinical care. mLearning provides mobile platforms for educational support for health professionals. Finally, health financing applications are those that facilitate the use of smart cards or vouchers for mobile payments. The purpose of this article is to explain the context for regulation, identify implications of regulation, and discuss future perspectives of mHealth applications.

Of the myriad of mobile medical applications available to clinicians and their patients, a small subset has been recommended for regulatory oversight in the United States. These are mobile applications whose intended use is similar to that of an existing medical device. Vos and Parker [4] state 'Medical devices by their very nature have the potential to present a hazard - to be a source of harm in normal use, and more so if misused. Regulations are therefore a necessary instrument to safeguard users from undue and unnecessary risks and are based on the principle of mitigating, to an acceptable level, the potential of a device to cause harm.' They further explain that the purpose of regulation is not only geared toward manufacturers, but also to provide guidelines to support product development in a manner which results in an appropriate risk-benefit ratio.

Since publication of 'Crossing the Quality Chasm', a report by the Institute of Medicine (IOM) that focused on creating health system solutions to mitigate medical error, health information technology (Health IT) has been heralded as a vehicle to enhance patient safety and provide patient-centered, timely, efficient, effective, and equitable care [5]. As the United States has moved toward the development of a national Health IT infrastructure, over 1,500 mobile medical applications have been developed to assist both patients and their clinicians in managing care [6]. The growing use of these applications, as well as the potential risks that those functioning as medical devices may pose to public health, prompted the United States Food and Drug Administration (FDA) to issue draft guidance concerning the regulation of mobile medical applications in July 2011 [7]. The role of the FDA is to protect consumers and enhance public health through maximizing compliance with regulated products and minimizing risk. The FDA has regulated medical devices for decades and is proposing to extend these regulations for mobile medical applications that function as medical devices.

## Discussion

Several types of mobile medical applications are proposed to be included in regulations (Table 1). One such example is an application used as an extension of a device such as a remote display of data from a bedside monitor. It would be important to ensure that the displays are consistent in the information provided to the clinician. A second group of mobile medical applications that would fall under regulations are those that use attachments, display screens, or sensors similar to an existing medical device. Included in this category are applications which attach a mobile device to a glucose strip reader. Once again, accuracy is necessary for appropriate diagnosis and intervention. Finally, mobile applications that are designed to assist diagnosis or treatment using specific patient data, such as calculation of medication dosage, will require regulation. Over- or under-dosing may result in serious patient harm. Thus, those applications that are targeted for regulation perform the same function as a currently regulated device, and could cause harm if they fail to function as intended.

**Table 1:** Mobile Medical Applications for which FDA will apply regulatory oversight

Description	Examples
Mobile applications that are an extension of one or more medical device(s) or displaying, storing, analyzing, or transmitting patient-specific medical device data	Remote display of data from bedside monitors Display of previously stored EEG waveforms Display of medical images directly from a Picture Archiving and Communication System (PACS) server Control of inflation/deflation of a blood pressure cuff Control of the delivery of insulin by an insulin pump

Mobile applications that transform the mobile platform into a medical device by using attachments, display screens, or sensors or by including functionalities similar to those of currently regulated medical devices	Attachment of a transducer to a mobile platform to function as a stethoscope Attachment of a blood glucose strip reader to a mobile platform to function as a glucose meter Attachment of electrocardiograph (ECG) electrodes to a mobile platform to measure, store, and display ECG signals

Mobile applications that allow the user to input patient-specific information and through the use of formulae or processing algorithms, output a patient-specific result, diagnosis, or treatment recommendation to be used in clinical practice or to assist in making clinical decisions	Mobile applications that provide a questionnaire for collecting patient-specific lab results and either: (1) compute the prognosis of a particular condition or disease; (2) perform calculations that result in an index or score; (3) calculate dosage for a specific medication or radiation treatment; or (4) provide recommendations that aid a clinician in making a diagnosis or selecting a specific treatment for a patient

Adapted from: **Draft Guidance for Industry and Food and Drug Administration Staff - Mobile Medical Applications **http://www.fda.gov/medicaldevices/deviceregulationandguidance/guidancedocuments/ucm263280.htm

The FDA defines a mobile medical application as one that meets the definition of a device ('... an instrument, apparatus, implement, machine, contrivance, implant, in vitro reagent' that is 'intended for use in the diagnosis of disease or other conditions, or in the cure, mitigation, treatment, or prevention of disease, in man...' or '... intended to affect the structure or any function of the body of a man or other animals...') and is either 'used as an accessory to a regulated medical device or transforms a mobile platform into a regulated medical device'. This table summarizes information about the mobile medical applications that will be subjected to regulation by the FDA. The table describes the mobile applications of interest and then provides specific examples of devices that would fall under the regulation.

Obviously, those medical applications that are intended to be used as devices are a relatively small proportion of those available for use. There are several types of applications that are not being considered for regulation. First and foremost, those applications that are used as reference guides (such as an electronic text book) for clinicians [8-10] or health and wellness records (for example, diet and weight logs) for consumers [11-13] are not being considered for regulation. However, applications that might be used for dosage calculation [14,15] or digital imaging [16,17], do fall under the new regulation guidance. Applications that support office operations, or those that are generic aids like a magnifying glass that assist users but are not marketed for medical purposes are also not included under the regulation guidance.

The American Medical Informatics Association submitted feedback concerning regulation of mobile medical applications in a letter dated October 19, 2011. The themes of that response included clarification of definitions and use of terminology about medical mobile applications, the convergence of mobile and non-mobile technologies, the functionality of applications, and the evolution of patient care delivery processes and payment methods [18].

Another concern related to the potential of mobile health application regulations pertains to limiting innovation and discovery. Several implications of regulation from the perspective of a developer include additional cost, lead time for production, complexity, and paperwork [19]. For researchers, exemptions are possible, but the fear is that innovation would be curtailed due to the expanded length of time required to take a product to market.

Recently, safety issues concerning the use of Health IT have begun to surface, prompting the Office of the National Coordinator to commission an analysis by the Institute of Medicine (IOM) [20]. While no safety issues with regard to medical mobile applications emerged in their review, they cautioned that over-reliance on these applications may create barriers for consumers who have limited Internet connectivity and/or health literacy resulting in an unintended exacerbation of health access and disparity issues. Further, there are additional concerns about maintaining health data in a confidential manner across mobile computing platforms. In order to track safety concerns, the IOM report contains a recommendation for the Department of Health and Human Services to 'establish a mechanism for both vendors and users to report health IT-related deaths, serious injuries, or unsafe conditions' [20].

Similar issues are noted in developing nations. While penetration of mobile phone services is increasing, several issues of web-enabled applications such as cost, connectivity, coverage, low literacy, and high diversity of users emerge. Even though opportunities for mobile medical applications in developing countries seem profound, there is lack of evidence to support widespread use without careful testing and evaluation [21]. In fact, a recent report recommends creation of a global infrastructure for mobile medical applications to provide 'common standards and guidelines, and serves as a repository for shared resources and best practices' [22]. Specifically, evaluation of mobile medical applications should be strengthened to use methods such as comparison studies and cost-benefit studies in which standardized indicators, replicable study design, and adequate sample sizes are employed to assess not only the technology, but the impact of the technology on health [23]. The International Organization for Standardization recently released a new standard for medical devices which incorporates a risk assessment framework to establish technical or process standards [24]. Mobile medical applications which function as medical devices should be subject to the same standards in development, manufacturing, and use.

## Conclusions

Deliberations about the appropriateness of regulation of mobile medical applications must take a patient-centered approach. The FDA has provided initial guidance on their perspective. With the broader contributions of members of the healthcare and informatics communities, from both private and public sectors, regulations will likely be appropriate and reasonable, provided those regulations are applied to mobile medical applications that present potential risk for public health. The Hippocratic notion of 'do no harm' should be of primary consideration. Moving forward, it will be necessary to achieve a balance for ensuring patient safety while supporting innovation in the development and use of mobile medical applications.

## Abbreviations

FDA: United States Food and Drug Administration; Health IT: Health Information Technology; IOM: Institute of Medicine; mHealth: Mobile health; SMS: Short messaging service (more commonly known as text messaging).

## Competing interests

The author declares that they have no competing interests.

## Author information

AB is professor and Associate Dean for Clinical and Community Affairs at the University of Colorado College of Nursing. She is a fellow in the American Academy of Nursing, president of the National Nursing Centers Consortium, and a member of the American Medical Informatics Association.

## Pre-publication history

The pre-publication history for this paper can be accessed here:

http://www.biomedcentral.com/1741-7015/10/46/prepub
